# Molecular and Biochemical Characterization of Salt-Tolerant Trehalose-6-Phosphate Hydrolases Identified by Screening and Sequencing Salt-Tolerant Clones From the Metagenomic Library of the Gastrointestinal Tract

**DOI:** 10.3389/fmicb.2020.01466

**Published:** 2020-07-07

**Authors:** Yanxia Yang, Yunjuan Yang, Qin Fan, Zunxi Huang, Junjun Li, Qian Wu, Xianghua Tang, Junmei Ding, Nanyu Han, Bo Xu

**Affiliations:** ^1^School of Life Sciences, Yunnan Normal University, Kunming, China; ^2^Engineering Research Center of Sustainable Development and Utilization of Biomass Energy, Ministry of Education, Kunming, China; ^3^Key Laboratory of Yunnan for Biomass Energy and Biotechnology of Environment, Kunming, China

**Keywords:** trehalose-6-phosphate hydrolase, salt tolerance, gastrointestinal tract microbe, metagenomic library, high-throughput sequencing

## Abstract

The exploration and utilization of microbial salt-tolerant enzymatic and genetic resources are of great significance in the field of biotechnology and for the research of the adaptation of microorganisms to extreme environments. The presence of new salt-tolerant genes and enzymes in the microbial metagenomic library of the gastrointestinal tract has been confirmed through metagenomic technology. This paper aimed to identify and characterize enzymes that confer salt tolerance in the gastrointestinal tract microbe. By screening the fecal metagenomic library, 48 salt-tolerant clones were detected, of which 10 salt-tolerant clones exhibited stronger tolerance to 7% (wt/vol) NaCl and stability in different concentrations of NaCl [5%–9% (wt/vol)]. High-throughput sequencing and biological information analysis showed that 91 potential genes encoded proteins and enzymes that were widely involved in salt tolerance. Furthermore, two trehalose-6-phosphate hydrolase genes, namely, *tre_P2* and *tre_P3*, were successfully cloned and expressed in *Escherichia coli* BL21 (DE3). By virtue of the substrate of *p*-nitrophenyl-α-D-glucopyranoside (*p*NPG) which can be specifically hydrolyzed by trehalose-6-phosphate hydrolase to produce glucose and *p*-nitrophenol, the two enzymes can act optimally at pH 7.5 and 30°C. Steady-state kinetics with *p*NPG showed that the *K*_*M*_ and *K*_*cat*_ values were 15.63 mM and 10.04 s^–1^ for rTRE_P2 and 12.51 mM and 10.71 s^–1^ for rTRE_P3, respectively. Characterization of enzymatic properties demonstrated that rTRE_P2 and rTRE_P3 were salt-tolerant. The enzymatic activity increased with increasing NaCl concentration, and the maximum activities of rTRE_P2 and rTRE_P3 were obtained at 4 and 3 M NaCl, respectively. The activities of rTRE_P2 increased by approximately 43-fold even after 24 h of incubation with 5 M NaCl. This study is the first to report the identification as well as molecular and biochemical characterization of salt-tolerant trehalose-6-phosphate hydrolase from the metagenomic library of the gastrointestinal tract. Results indicate the existence of numerous salt-tolerant genes and enzymes in gastrointestinal microbes and provide new insights into the salt-tolerant mechanisms in the gastrointestinal environment.

## Introduction

Salt-tolerant bacteria mainly live in extreme salt-rich environments. These bacteria adapt to the special external environmental pressure by producing numerous salt-tolerant genes and enzymes. Exploring and utilizing microbial salt-tolerant enzymatic and genetic resources are of great significance in the study of biological adaptation to extreme environments and for broadening the application of salt-tolerant enzymes and genes in the field of biotechnology.

A large number of salt environments exist in natural habitats, where halophilic or salt-tolerant microorganisms are widely distributed. Researchers use pure culture techniques to obtain various salt-tolerant genes and enzymes from halophilic or salt-tolerant microorganisms such as *IoXyl2p* gene (Han et al., 2020), *pprI* gene (Hossein Helalat et al., 2019), α-amylase (Deutch and Yang, 2020), and β-xylosidase (Xu et al., 2019). However, the number of salt-tolerant genes and enzymes is still lower than that of salt-tolerant bacteria isolated. With the advent of the post-genome era, the emergence of metagenomic technology addresses the problem of microbial separation and culture, expands the utilization of microbial resources, and provides a new research strategy for discovering new salt-tolerant functional genes and enzymes.

The gastrointestinal tract is considered the densest microbial ecosystem on the planet (Whitman et al., 1998; Egert et al., 2006) and is a huge resource for exploring biocatalysts. Microbes inhabiting in the gastrointestinal tract are exposed to stresses, such as acids, bile salts, and osmosis pressure (Louis and O’Byrne, 2010). Hyperosmosis gastrointestinal cavity (equivalent to 0.3 M) (Alvarez-Ordonez et al., 2011) and dietary changes (De Dea Lindner et al., 2007) can lead to the production of osmotic pressure in the gastrointestinal tract. In this external environmental pressure, some special genes and enzymes of microorganisms may be activated to adapt to pressure.

Few studies have investigated on salt-tolerant genes and enzymes from the metagenome of the gastrointestinal tract. Since 2012, Culligan et al. (2012) obtained salt-tolerant clones from the metagenomic library of human gut microbiomes as well as salt-tolerant genes and enzymes, such as *galE*, *murB*, *mazG* (Culligan et al., 2012), *stlA* (Culligan et al., 2013), *sdtR* (Culligan et al., 2014a), and *brpA* (Culligan et al., 2014b). *galE* encodes UDP-glucose 4-epimerase. *murB* encodes UDP-*N*-acetylenolpyruvoyl glucosamine reductase. *mazG* encodes the MazG family of proteins (nucleoside triphosphate pyrophosphohydrolase), which can delay the procedural death of cells. The *stlA* gene is a salt-tolerant gene that is unique to the human gut; this gene can gain a competitive advantage in a high-salt environment by obtaining adjacent genes through lateral gene transfer (LGT) events. *sdtR* encodes putative transcriptional regulators. The *brpA* gene belongs to the brp/blh family of β-carotene 15,15′-monooxygenase, which encodes a membrane protein. Verma et al. (2018) screened salt-tolerant clones, namely, SR6 and SR7, from the human fecal metagenomic library, performed subclone analysis, and found that *TMSRP1*, *ABCTPP*, and *TLSRP1* are associated with salt tolerance. Ilmberger et al. (2012) obtained salt-tolerant hydrolase from the gastrointestinal microbial metagenome (celA84 in the GH5 family can retain 50% relative activity after 34 days in 4 M NaCl). Hence, gastrointestinal microorganisms contain abundant salt-tolerant genes and enzymes that can be obtained through metagenomic technology.

*Nycticebus pygmaeus* is a low-class primate and omnivorous animal that inhabits tropical rainforests, seasonal rainforests, and subtropical evergreen broad-leaved forests. *Bos frontalis* is a rare bovine species living in free-range conditions within tropical rainforest ecosystems which lie merely in some regions in India, Bangladesh, Bhutan, China, and Myanmar (Mondal et al., 2004). The animal is believed to be domesticated for as long as 8000 years, during which common salt was exclusively provided to the animal by local farmers as an additional feed (Das et al., 2011). This study screened salt-tolerant clones from the constructed fecal metagenomic library of *N. pygmaeus* and *B. frontalis* (Xu et al., 2014; Dong et al., 2016). We analyzed potential salt-tolerant genes and enzymes through high-throughput sequencing and bioinformatics technology. We then selected trehalose-6-phosphate hydrolase for cloning, heterologous expression, purification, and characterization. Recombinant rTRE_P2 and rTRE_P3 were found to be involved in salt tolerance. This study lays a theoretical basis for exploring the salt-tolerance mechanism and for the development and utilization of salt-tolerant genes and enzymes in gastrointestinal microorganisms.

## Materials and Methods

### Materials, Bacterial Strain, and Culture Conditions

Taq DNA polymerase, restriction endonucleases, and PCR reagents were obtained from Takara (Beijing, China). In-Fusion HD Cloning Kit (Takara Beijing, China), plasmid pEASY-E2, and *E. coli* BL21(DE3) competent cells (TransGen, Beijing, China) were adopted in the cloning process. The purification was performed with Ni-NTA Agarose resin (Qiagen, Hilden, Germany). *p*-Nitrophenyl-α-D-glucopyranoside (*p*NPG), *p*-nitrophenyl-β-D-galactopyranoside, *p*-nitrophenyl-β-D-glucopyranoside, *p*-nitrophenyl-α-D-mannopyranoside, and 2-nitrophenyl-β-D-glucopyranoside were supplied by Shanghai Yuanye Bio-Technology (China). The vector pCC1FOS and *E. coli* EPI300 were purchased from Epicentre. The remaining chemicals reagents were of analytical grade except for those specified. The cultivation environment of the strains *E. coli* is at 37°C in the Luria–Bertani (LB) liquid medium, in which the salt concentrations can reach their final stages, encompassing the salt showed in the normal LB medium. In order to select certain plasmids, 12.5 μg/ml chloramphenicol (Cm) or ampicillin (100 μg/ml) was used as an alternative of the medium. Previously constructed fecal microbial metagenomic libraries of *N. pygmaeus* and *B. frontalis* were used (Xu et al., 2014; Dong et al., 2016).

### Screening of Salt-Tolerant Clones

Augmented fecal microbial metagenomic library culture was inoculated into an LB broth complemented with 12.5 μg/ml chloramphenicol (Cm) and 7% (wt/vol) NaCl (1 ml of the former into 20 ml of the later). The flakes were nurtured at 37°C for 24 consecutive hours and unceasing shaking condition at 180 rpm. On top of that, new flasks with 20 ml of the identical components received 1 ml of the culture. The enrichment process for salt-tolerant clones was done for three times. The agar with the same medium was covered by the cultures. Noticeable colonies then emerged on the plates following the incubation for 72 h at 37°C. The isolated colonies were analyzed by an enzyme labeling apparatus. The optical density value (OD_600_) of the culture was determined after activation of salt-tolerant clones. The activated culture (20 μl) was shifted to 20 ml of the LB liquid medium [including 12.5 μg/ml Cm and 7% (wt/vol) NaCl] and nurtured for 24 h, 37°C, 180 rpm. The optical density value (OD_600_) was recorded using 200 μl of the culture. *E. coli* EPI300-C culture containing the empty pCC1FOS was set as the control group. Clones with high optical density value were selected for study of tolerance to different salt levels [5%–9% (wt/vol) NaCl].

### Fosmid DNA Extraction

Bacterial culture (5 ml) was brought into existence after the cultivation of 12.5 μg/ml Cm for a night. Near 1 ml of the culture was added to the broth with 4 ml fresh LB. Moreover, 5 μl of the 1000 copy control induction solution (Epicentre Biotechnologies), together with 12.5 μg/ml Cm, was put in the culture. The mixture was cultured at 37°C for 5 h with vigorous shaking (200–250 rpm) to ensure maximum aeration. All 5-ml stimulated cultures generated cells after the 1-min centrifugation at 12000 rpm. ZR BAC DNA Miniprep Kit (Zymo Research) was used to extract fosmid based on the manufacturer’s directions.

### High-Throughput Sequencing and Bioinformatics Analysis

The fosmid DNA of salt-tolerant clones was sequenced with the Illumina Solexa Genome Analyzer platform. Clean data were obtained by removing the low-quality base at both ends of the raw data and removing the sequences containing the adapter and consisting of a low average base mass, multiple N, and very short length. The sequences were assembled using SPAdes (v3.11.1) to obtain high-quality scaffold fragments. Scaffolds with length > 300 bp were retained for bioinformatics analyses. Megablast against the NCBI plasmid database^1^ was adopted to compare and filter the results in line with identity ≥ 30% and math length ≥ 200. cBar (v1.2) and PlasmidFinder (v1.3) were used to identify plasmids and select scaffold fragments as plasmid for genetic prediction. Prodigal (v2.6.3) was used as prediction software. Gene function annotation and secretory protein prediction analysis were carried out using Blast (v2.7.1 plus) and Diamond (v0.9.10) against Gene Ontology (GO), Cluster of Orthologous Groups of Protein (COG), Kyoto Encyclopedia of Genes and Genomes (KEGG), and nonredundant protein databases (NR). Low-reliability comparison results were filtered out according to identity, e-value, and score values. The optimal results were filtered as functional annotation results for the gene.

### Cloning of Trehalose-6-Phosphate Hydrolase

*Tre_P2* and *tre_P3* were identified in salt-tolerant clones 1A and 5_1_1 in *N. pygmaeus*, and *tre_P4* was identified in salt-tolerant clone 21_9A in *B. frontalis* by high-throughput sequencing. The plasmid DNA of clones 1A, 5_1_1, and 21_9A were used as template for PCR amplification. The primer pairs are as follows: *tre_P2*-F: 5′-TAA GAA GGA GAT ATA CAT ATG GAA TTG ATG AGT GAA CAA GAC TGG-3′ and *tre_P2*-R: 5′-GTG GTG GTG GTG GTG CTC GAG CTT CGC CGT TTC AAC CAC AAT TGC-3′. *tre_P3*-F: 5′-TAA GAA GGA GAT ATA CAT ATG GAA TTG ATG GAA TTG ATG AGT GAA CAA GAC TGG-3′, and *tre_P3*-R: 5′-GTG GTG GTG GTG GTG CTC GAG GTG GTG GTG GTG GTG GTG-3′, *tre_P4*-F: TAA GAA GGA GAT ATA CAT ATG GAA TTG ATG GAA TGG CAA GTA GGT ATC, and *tre_P4*-R: GTG GTG GTG GTG GTG CTC GAG ATA TGA CTT ACT AAT AAC TAT CGC (primers highlighted with underlines stand for *Nde* I and *Xho* I sites, specifically). The cloning process of amplified fragments into the pEASY-E2 vector was done after PCR. The verification of gene sequence was achieved through DNA sequencing.

### Expression and Purification

*E. coli* BL21 (DE3) transformed with pEASY-E2 containing *tre_P2* or *tre_P3* fragments was incubated overnight on the LB broth supplemented with 100 μg/ml ampicillin in a shaking incubator at 180 rpm and 37°C. What followed was the shifting process of the culture (1 ml) to new flasks with the same portion of 200 ml. 0.7 mM isopropyl-D-thiogalactopyranoside (IPTG) was used to induce thriving cells in the optimum density of 0.6 at 600 nm, which further grew for 20 h at 20°C, 160 rpm. Cells were gathered after centrifugation (6000 × *g* for 8 min) at 4°C, suspended in binding buffer (0.5 M NaCl, 20 mM imidazole, 20 mM Tris–HCl, pH 8.0), and the disrupted with a high-pressure cell disruptor. The centrifugal process was then done to the cell lysate (12000 × *g* and 4°C for 20 min). For the analysis of the subsequent supernatant, sodium dodecyl–sulfate polyacrylamide gel electrophoresis (SDS-PAGE) and enzyme assay were adopted. By virtue of the Ni-NTA His⋅Bind column and immobilized metal-affinity chromatography, the enzyme was purified. A Ni-NTA His⋅Bind column, which was equilibrated earlier using binding buffer, was adopted to carry the sample. The column used was washed with binding buffer and washing buffer (concentration: 0.5 M NaCl, 80 or 100 mM imidazole, 20 mM Tris–HCl, pH 8.0). Eluting buffer (500 mM imidazole, 0.5 M NaCl, 20 mM Tris–HCl, pH 8.0) was used to elute the bound protein. Those fractions carrying the recombinant protein were gathered and preserved at −20°C.

### Enzyme Assay and Characterization

The following experiments were repeated three times.

Trehalose-6-phosphate hydrolase hydrolyzes *p*NPG to produce glucose and *p*-nitrophenol, which is deemed as a specific process as *p*NPG does not hydrolyze itself in the absence of trehalose-6-phosphate hydrolases, nor will it be hydrolyzed by the periplasmic trehalase or maltodextrin-degrading enzymes (Rimmele and Boos, 1994). *p*NPG was employed, on which rTRE_P2 and rTRE_P3 activities were testified, adopting the method conveyed by Chuang et al. (2012). The combination for further reaction was with 5 mM *p*NPG, 100 mM NaCl, and 50 mM HEPES–NaOH buffer (pH 7.5) and a certain amount of the enzyme reagent. First cultivated for 10 min at 30°C, the reaction was then boiled for 5 min, which in the end caused it to cease. Centrifuge was used to separate the sample, and A410 of the supernatant was registered. Delineation of a single unit of rTRE_P2 and rTRE_P3 activities was considered as related to the quantity of the enzyme capable of discharging 1 μmol *p*-nitroaniline per minute at 30°C.

Temperature influence on the activities of rTRE_P2 and rTRE_P3 was reviewed at the range of 0°C–70°C. On the substrate of *p*NPG operated the reactions in standard conditions. In a bid to study the influence of temperature on enzyme stability, a control group containing different temperatures (37°C, 45°C, and 50°C) was conducted on rTRE_P2 and rTRE_P3 for an hour. Regardless of the designated temperature, the activity of the residual enzyme was circumscribed in a 10-min span during the given time.

The pH with which the activities of rTRE_P2 and rTRE_P3 were examined was from 3 to 10. The activities of rTRE_P2 and rTRE_P3 were assayed at 30°C in citric acid–Na_2_HPO_4_ buffer (pH 3–5) and HEPES-NaOH buffer (6–12). The pH stability changed with different status in incubating the purified enzyme in different buffers during the 1-h period at 30°C, for which the untreated enzyme solution was adopted as means of controlling.

Determination of the effect of different NaCl concentrations on rTRE_P2 and rTRE_P3: different concentrations of NaCl solution (0.5–5.0 M) were prepared with HEPES–NaOH (pH 7.5) containing 5 mM *p*NPG, and residual enzyme activity was determined at the optimum condition, with no NaCl enzymatic reaction added as the control group. Determination of NaCl stability of rTRE_P2 and rTRE_P3: the purified recombinant rTRE P2 and rTRE P3 were added into 0.5–5 M NaCl solution and treated for 1 or 24 h at 30°C, respectively. The residual enzyme activity was determined at the optimum pH and optimum temperature, and the enzymatic reaction without NaCl treatment under the same conditions was used as the control group.

In evaluating the effects of metal ions and chemicals on the activities of rTRE_P2 and rTRE_P3 (which include GuHCl, KCl, NaCl, FeSO_4_, CoCl_2_, iodoacetic acid, pig bile salt, Tween 80, Triton X-100, FeCl_3_, ZnSO_4_, CTAB, EDTA, NiSO_4_, MnSO_4_, AgNO_3_, β-mercaptoethanol, DTT, EGTA, CuSO_4_, PEG20000, SDS, NBS, MgCl_2_, HgCl_2_, AlCl_3_, LiCl, PbCl_2_, and CaCl_2_), a 10-min incubation process was carried out on purified enzyme aliquots at 30°C in 50 mM HEPES–NaOH buffer (pH 7.5). Residual activity was assessed according to standard assay situations. Enzymatic activity in exclusion of the metal ions and chemicals beyond the experiment was supposed as 100%.

In environments with a temperature of 30°C and a pH of 7.5, multiform *p*-nitrophenyl derivatives were utilized to delve the specificities brought from the substrate of rTRE_P2 and rTRE_P3. The substrate composites applied in the experiment involved *p*NPG, *p*-nitrophenyl-β-D-galactopyranoside, *p*-nitrophenyl-β-D-glucopyranoside, *p*-nitrophenyl-α-D-mannopyranoside, and 2-nitrophenyl-β-D-glucopyranoside. The substrates used to incubate the purified enzyme also include glucose, glucose-6-phosphate, trehalose, and trehalose-6-phosphate [0.5% (wt/vol)], and the environment in which it grew for 2 h was in 50 mM HEPES–NaOH buffer (200 μl, pH 7.5) at 30°C. The enzymatic fruits (2 μl) here were located on a silica plate. Water, acetic acid, and 1-butanol were mixed (1:1:2, vol/vol/vol) for the operation of thin-layer chromatography (TLC). The specimen was dipped into 50 ml of acetone containing 1 g of diphenylamine, added with 1 ml of phenylid and 5 ml of 85% phosphorous acid for 30 s, and heated at 120°C for 15 min.

To study the steady-state kinetics, the samples were monitored at 30°C for 12 min (first-order reaction time) in a reaction buffer with *p*NPG concentrations of 0.5–10 mM. Moreover, based on the least-square fits of initial rates, kinetic parameters were evaluated, making their functions of substrate concentration stand out. The figuration of *K*_*M*_ and *K*_*cat*_ values was achieved through gauging the gradient of the linear part of the Michaelis–Menten plot.

## Results

### Screening of Salt-Tolerant Clones

**FIGURE 1:**
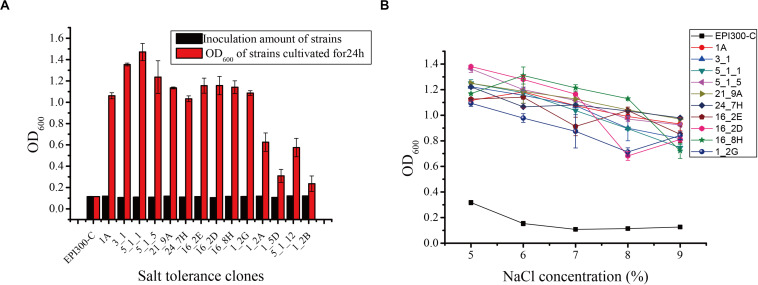
**(A)** Screening of some salt-tolerant clones of the fosmid library. **(B)** Salt tolerance of some salt-tolerant clones against different salt stress levels. Error bars represent the mean ± SD (*n* = 3).

Seventeen and 31 salt-tolerant clones were detected from the metagenomic fosmid libraries of *N. pygmaeus* and *B. frontalis*, respectively. The growth of partial salt-tolerant clones is shown in Figure 1A. After 24 h of culture, the OD_600_ of 48 salt-tolerant clones was 0.236–1.471, indicating that these strains were salt-tolerant. Clone 5_1_1 had a high ability to resist 7% (wt/vol) NaCl, and clone 1_2B has the weakest tolerance to 7% NaCl (wt/vol). The control *E. coli* EPI300-C strain was cultured in 7% (wt/vol) NaCl medium, and no significant change was noted. Hence, microbial salt-tolerant gene fragments may have been inserted into the pCC1FOS vector. Ten clones (1A, 3-1, 5_1_1, and 5_1_5 from the *N. pygmaeus* gastrointestinal tract and 21_9A, 24_7H, 16_2E, 16_2D, 16_8H, and 1_2G from the *B. frontalis* gastrointestinal tract) with high salt tolerance were selected for the study of tolerance to different salt levels (Figure 1B). With increasing NaCl concentration (5% to 9%), the OD_600_ of the strains remained basically stable, showing significant salt tolerance compared with the controls.

### High-Throughput Sequencing and Functional Classification of Unigene

To obtain salt-tolerant genes and enzymes from the gastrointestinal tract of microbes, we sequenced the fosmid DNA of 10 salt-tolerant clones by high-throughput sequencing. We obtained 100,909,958 raw reads with a total of 15,136,493,700 bps. After trimming, 94,533,758 clean reads remained. Low-quality reads and adapter sequence were filtered. There were 323,181 bp effective sequences (average length: 5,572.09 bp) produced from the gastrointestinal tract of *N. pygmaeus* and 898,646 bp effective sequences (average length: 3,907.16 bp) from the gastrointestinal tract of *B. frontalis* (Supplementary Table S1).

Gene Ontology analysis showed that most genes are associated with biological processes including metabolic and cellular processes (Supplementary Figure S1). Clones 3_1 and 1A, but not clone 5_1_5, are related to metabolic processes alone. Only clone 21_9A is associated with cellular components, which include membrane parts. Most genes, including clones 3_1 and 16_2D, are involved in molecular functions, such as binding but not catalytic activity.

**FIGURE 2:**
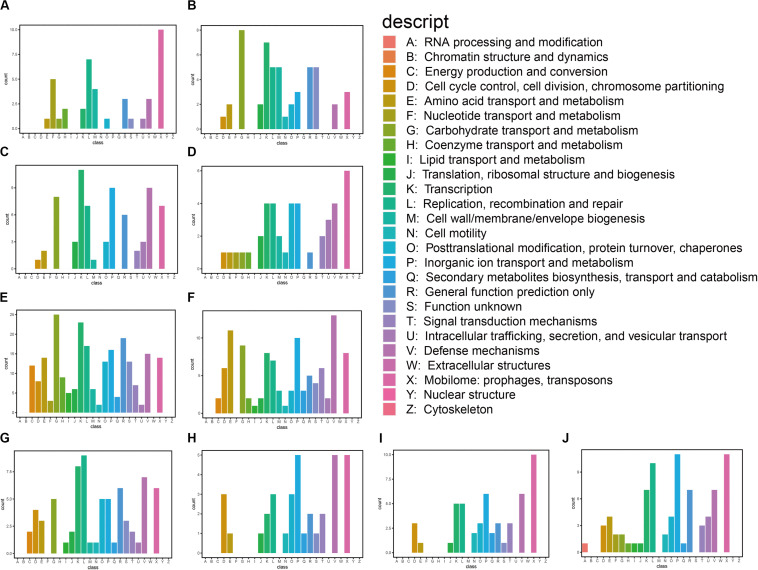
Functional classification of annotated transcripts by COG. **(A)** 3_1; **(B)** 1A; **(C)** 5_1_1; **(D)** 5_1_5; **(E)** 21_9A; **(F)** 24_7H; **(G)** 16_2E; **(H)** 16_2D; **(I)** 16_8H; and **(J)** 1_2G.

We also classified the predicted genes by aligning them to COG and KEGG databases. Based on the annotation against the COG database, these genes are mainly associated with carbohydrate transport and metabolism, transcription, replication, recombination and repair, inorganic ion transport and metabolism, and defense mechanism: mobilomes, prophages, and transposons (Figure 2). We found that the predicted functional genes in the gastrointestinal tract of *N. pygmaeus* were not involved in secondary metabolite biosynthesis, transport, and catabolism (Figures 2A–D), whereas the predicted functional genes in the gastrointestinal tract of *B. frontalis* were involved in such processes (Figures 2E–J). The contrasting finding may be due to dietary differences. The KEGG analysis of the salt-tolerant clones showed presumed components of carbohydrate metabolism, membrane transport, drug resistance, and nucleotide metabolism, which perhaps implicated diverse metabolic pathways related to salt tolerance (Supplementary Figure S2). Hence, salt-tolerant strains may have a specific membrane structure, transcriptional regulation process, high transport, and metabolic capacities.

### Screening and Analysis of Potential Salt-Tolerant Genes and Enzymes

We annotated genes to the Nr database. A total of 1,285 genes and enzymes of salt-tolerant clones were significantly annotated (data not shown). After comparative analysis, 91 genes and enzymes were assumed to be widely involved in salt resistance. These genes and enzymes include UDP-glucose 4-epimerase, general stress protein, transcriptional regulator, alcohol dehydrogenase, glucose-6-phosphate 1-dehydrogenase, trehalose-6-phosphate hydrolase, transporters, etc. (Supplementary Table S2). Fifty-two potential salt-tolerant genes and enzymes were identified from the gastrointestinal tract of *B. frontalis*, which are more than the genes and enzymes from the gastrointestinal tract of *N. pygmaeus* (39 genes). UDP-glucose 4-epimerase, catalase, and cadmium transporter were only detected in the gastrointestinal tract of *N. pygmaeus*. Trehalose-6-phosphate hydrolase is considered irreducible in the assimilation of trehalose (Chuang et al., 2012), with which the inactivating or denaturizing of proteins and cellular membranes can be prevented even in conditions such as desiccation, hypohydration, torridness, coldness, and oxidation (Elbein et al., 2003). Thus, we chose trehalose-6-phosphate hydrolase for further study. In addition, we found multiple functional genes that are resistant to other stresses, such as glutaredoxin and cold-shock proteins.

### Cloning and Sequence Analysis of Trehalose-6-Phosphate Hydrolase

**FIGURE 3:**
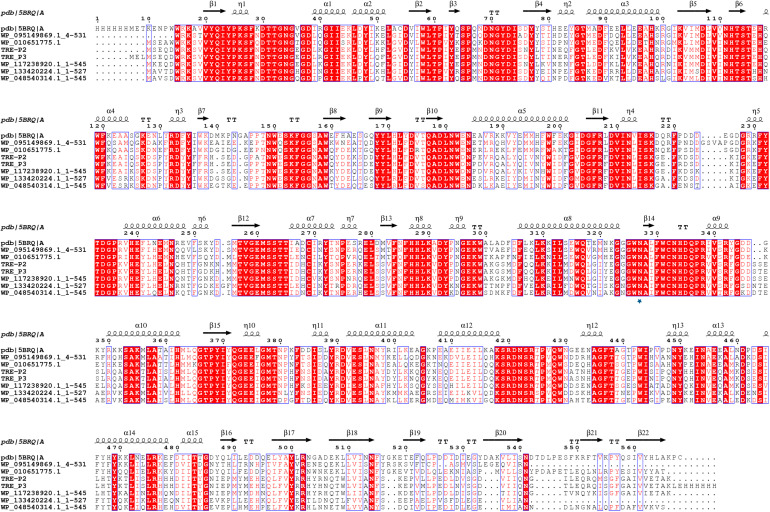
Multiple-sequence alignment analysis of TRE_P2 and TRE_P3. Glycoside Hydrolase Family 13 of 5BRQ Chain A (GenBank: AE015928; PBD: 3D3A) from *B. licheniformis* was used as the secondary structural template. WP_095149869.1:4-531, WP_010651775.1, WP_117238920.1:1-545, WP_133420224.1:1-527, and WP_048540314.1:1-545 are from *Bacillus sp. BO*, *Oceanobacillus massiliensis*, *Staphylococcus pasteuri*, *Macrococcus canis*, and *Staphylococcus sciuri*, respectively.

PCR amplification proved unsuccessful for *tre_P4* from clone 21_9A in the gastrointestinal tract of *B. frontalis*, possibly owing to special structures of this gene. As such, *tre_P2* and *tre_P3* from salt-tolerant clones 1A and 5_1_1 in the gastrointestinal tract of *N. pygmaeus* were chosen for further investigation. *tre_P2* (1638 bp) and *tre_P3* (1644 bp) were obtained through PCR amplification, and the nucleotide sequence was preserved in GenBank (MN830270, MN830271). SignalP server^2^ was adopted, as a means of testing signal peptide, to analyze the translated amino acid sequence in the gene. The results indicate that TRE_P2 and TRE_P3 have no signal peptides. The homology search conducted on NCBI through the BLASTP tool suggested that TRE_P2 had the highest sequence identity (100%) and TRE_P3 had the highest sequence identity (99.82%) to trehalose-6-phosphate hydrolase from *Staphylococcus warneri* (WP_124228261) and *Staphylococcus warneri* (WP_058709819), respectively. However, these proteins were not characterized. The deduced amino acid sequences of TRE_P2 and TRE_P3 were compared with those of the available trehalose-6-phosphate hydrolase from different microorganisms by using CLUSTALX (2.1) program and mapped by ESPript.^3^ From the results, we found a site Asn327 marked by a pentangle (Figure 3). The site is considered essential for binding to chloride ions according to Ong et al. (2014). Moreover, the findings may help explain why TRE_P2 and TRE_P3 confer salt tolerance.

**FIGURE 4:**
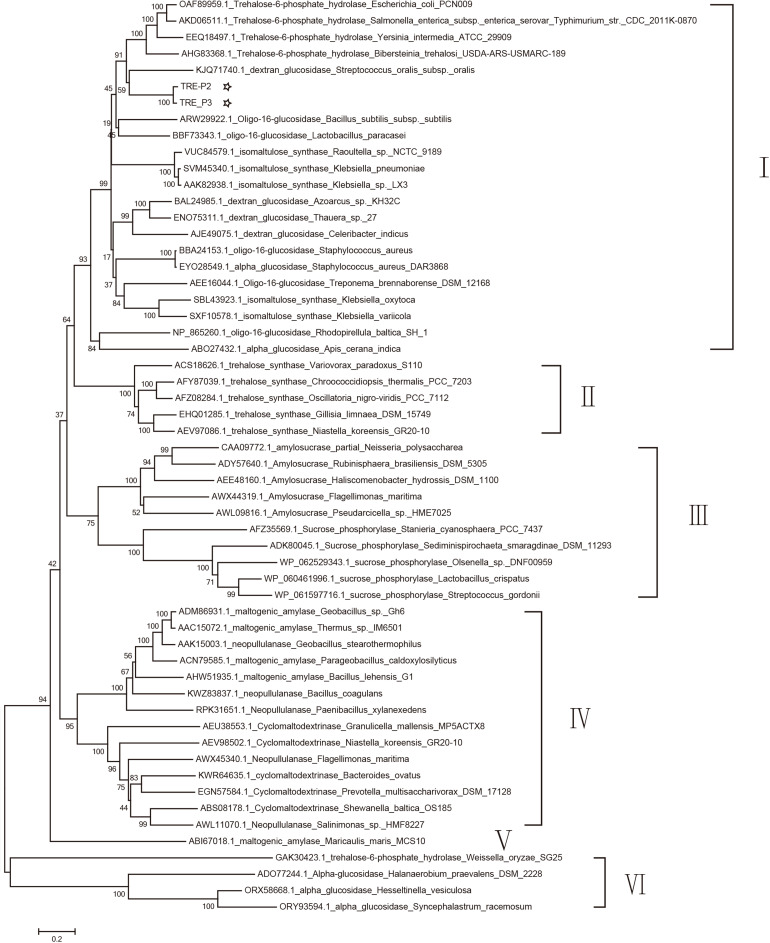
Phylogenetic tree analysis of oligo-1,6-glucosidase, α-glucosidase, dextran glucosidase, trehalose-6-phosphate hydrolase, amylosucrase, sucrose phosphorylase, isomaltulose synthase, trehalose synthase, cyclomaltodextrinase, maltogenic amylase, and neopullulanase which originated from various microorganisms homologous to TRE_P2 and TRE_P3 by the neighbor-joining method.

A dendrogram was constructed in accordance with amino acid sequences to attest the evolution correlation of TRE_P2 and TRE_P3 with other disclosed oligo-1,6-glucosidase, α-glucosidase, dextran glucosidase, trehalose-6-phosphate hydrolase, amylosucrase, sucrose phosphorylase, isomaltulose synthase, trehalose synthase, cyclomaltodextrinase, maltogenic amylase, and neopullulanase that originated from various microorganisms. As shown in Figure 4, TRE_P2 and TRE_P3 were divided into one group and had a closer relationship to trehalose-6-phosphate hydrolase.

### Expression, Purification, and Molecular Size Determination of rTRE_P2 and rTRE_P3

The expression of rTRE_P2 and rTRE_P3 as an N-terminal His-tag fusion protein to characterize biochemical properties was conducted through the pEASY-E2 expression system, in which the T7 lac promoter in *E. coli* BL21 (DE3) was used as a controller. A high-pressure cell disruptor was adopted to break apart the cells collected. Following purification with the Ni-NTA column and assay using SDS-PAGE analysis, roughly 65 kDa molecular mass of the purified enzymes was gathered (Supplementary Figure S3). The comparative molecular masses of rTRE_P2 and rTRE_P3 were estimated to be about 65.21 and 65.67 kDa, respectively, proving them to be monomeric proteins.

### Biochemical Properties of the Recombinant Enzyme

**FIGURE 5:**
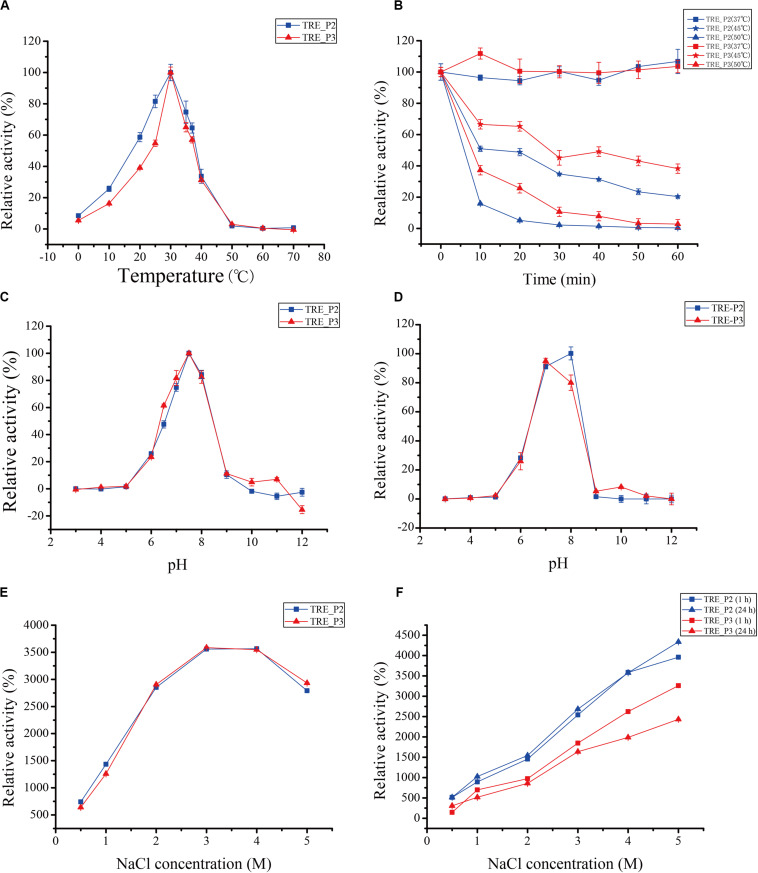
Effects of temperature, pH, and salts on rTRE_P2 and rTRE_P3 activities. **(A)** optimum temperature; **(B)** temperature stability; **(C)** optimum pH; **(D)** pH stability; **(E)** effect of NaCl; and **(F)** NaCl stability. Error bars represent the mean ± SD (*n* = 3).

The purified enzyme was tested at a temperature range of 0°C–70°C for 10 min to get its apparent temperature dependence. As indicated in Figures 5A,B, the enzymes reach their most dynamic status at 30°C and their most stable status at 37°C.

The optimum pH activity assay of rTRE_P2 and rTRE_P3 was performed under standard conditions but with different buffer components. In 50 mM HEPES–NaOH buffer, the optimum pH for rTRE_P2 and rTRE_P3, 7.5, exists (Figure 5C). At pH 10, the rTRE_P3 activity was reduced to 4.95% and rTRE_P2 completely lost its activity. Recombinant rTRE_P2 and rTRE_P3 were stable within the pH between 7 and 8 (Figure 5D).

Enzymatic activities of rTRE_P2 and rTRE_P3 were stimulated by NaCl, implying certain effect (Figure 5E). The enzymatic activity increased with increasing NaCl concentration, and the maximum activities of rTRE_P2 and rTRE_P3 were obtained at 4 and 3 M NaCl, respectively (approximately 35-fold). The activity also increased after 1 h of incubation with the involvement of 0.5–5 M NaCl, and this trend remained after 24 h. The activity of rTRE_P2 even increased by approximately 43-fold after incubation with 5 M NaCl after 24 h (Figure 5F).

Immersed in HEPES–NaOH buffer (50 mM, pH 7.5, 30°C) for 10 min, various chemicals were pre-incubated to examine their possible effects on the activities of rTRE_P2 and rTRE_P3. The activities were increased by GuHCl, KCl, NaCl, EDTA, β-mercaptoethanol, DTT, and EGTA but completely inhibited by PbCl_2_, CTAB, AgNO_3_, CuSO_4_, HgCl_2_, and ZnSO_4_ (Table 1). Recombinant rTRE_P2 and rTRE_P3 activities were slightly affected by iodoacetic acid, NBS, Triton X-100, PEG20000, and Tween 80. MgCl_2_ and MnSO_4_ increased the rTRE_P2 activity but inhibited the rTRE_P3 activity. The remaining ions significantly inhibited the activities of rTRE_P2 and rTRE_P3.

**TABLE 1:** Effects of various chemicals on relative activity of recombinant rTRE_P2 and rTRE_P3.

Chemicals (1 mM)	Relative activity of rTRE_P2	Relative activity of rTRE_P3
**Metal ions**		
KCl	110.54 ± 9.22%	122.77 ± 2.64%
NaCl	104.48 ± 4.21%	112.28 ± 2.08%
FeSO_4_	26.50 ± 5.57%	44.65 ± 0.84%
CoCl_2_	72.50 ± 5.43%	68.19 ± 0.66%
FeCl_3_	29.23 ± 1.07%	7.70 ± 0.66%
ZnSO_4_	0.65 ± 0.24%	1.46 ± 0.22%
NiSO_4_	72.58 ± 8.76%	42.25 ± 3.38%
MnSO_4_	111.08 ± 6.49%	70.31 ± 4.14%
AgNO_3_	0.32 ± 0.39%	−7.60 ± 5.72%
CuSO_4_	−0.42 ± 0.90%	−0.02 ± 0.42%
CaCl_2_	22.50 ± 7.83%	53.86 ± 3.85%
MgCl_2_	103.90 ± 9.23%	76.04 ± 2.77%
HgCl_2_	−0.45 ± 0.37%	−0.16 ± 0.06%
AlCl_3_	36.58 ± 16.09%	38.8 ± 4.47%
LiCl	53.28 ± 1.85%	58.4 ± 1.33%
PbCl_2_	−2.66 ± 4.32%	1.87 ± 0.95%
**Nonmetal ions**		
NBS	116.02 ± 3.34%	96.4 ± 2.01%
GuHCl	110.35 ± 13.92%	125.32 ± 3.11%
Iodoacetic acid	99.20 ± 10.91%	103.26 ± 1.53%
Pig bile salt	72.73 ± 6.74%	74.44 ± 4.96%
Tween 80	90.85 ± 7.39%	96.64 ± 6.03%
Triton X-100	85.34 ± 5.05%	99.26 ± 1.12%
CTAB	−0.06 ± 0.02%	0.26 ± 0.13%
EDTA	131.17 ± 6.51%	142.71 ± 2.13%
β-Mercaptoethanol	128.38 ± 0.70%	119.66 ± 1.67%
DTT	127.52 ± 2.35%	136.50 ± 2.84%
EGTA	126.21 ± 1.77%	120.66 ± 4.84%
PEG20000	108.99 ± 3.37%	94.89 ± 1.27%
SDS	11.17 ± 3.91%	4.64 ± 0.20%

Four various *p*-nitrophenyl derivatives were adopted in the examining of purified rTRE_P2 and rTRE_P3 substrate specificity. These enzymes hydrolyzed *p*NPG efficiently but not *p*-nitrophenyl-β-D-galactopyranoside, *p*-nitrophenyl-β-D-glucopyranoside, *p*-nitrophenyl-α-D-mannopyranoside, and 2-nitrophenyl-β-D-glucopyranoside. With *p*NPG as the substrate, the *K*_*M*_ and *K*_*cat*_ values of rTRE_P2 were 15.63 mM and 10.04 s^–1^, and those of rTRE_P3 were 12.51 mM and 10.71 s^–1^, respectively. TLC was then adopted to further assay rTRE_P2 and rTRE_P3 their specific substrate quality. As demonstrated in Supplementary Figure S4, no hydrolysis of trehalose was observed in the environment provided. It is believed that rTRE_P2 and rTRE_P3 have cleaved the trehalose-6-phosphate effectively and glucose and glucose-6-phosphate was thus generated.

## Discussion

Exploring salt-tolerant genes and enzymes in different environments has profound significance to reveal the salt-tolerant mechanism of microorganisms and direct their application in the high-salinity environment. At present, salt-tolerant enzymes and genes from marine bacteria (Zhang et al., 2017), soil (Petrovskaya et al., 2016), and gastrointestinal tract (Culligan et al., 2014b) as well as pond (Kapardar et al., 2010a), lake (Martinez-Martinez et al., 2013), biogas (Ilmberger et al., 2012), and wastewater treatment plants (Yang et al., 2016) have been reported. Salt-tolerant genes and enzymes are widely applied in high-salt food processing, seafood processing, washing, and other biotechnology fields with high salt concentrations (Margesin and Schinner, 2001). It is even more interesting that the gastrointestinal tract has its own unique salt-tolerant genes, which, through more sensitive and innovative screening assays, may stand out from salt-tolerant genes found in other environments when it comes to human-related biotech, medicine, or health researches (Culligan et al., 2013). The metagenomic technology explores the diversity and functions of microorganisms avoiding pure cultures and exhibits potential for discovering novel genes, developing new microbial active substances, and studying microbial community structure and function. In this study, we obtained 17 and 31 salt-tolerant clones from the metagenomic libraries of *N. pygmaeus* and *B. frontalis*, respectively. We selected ten clones with high salt tolerance for sequencing using high-throughput sequencing and function annotation.

Gene Ontology analysis showed that most genes in salt-tolerant clones were associated with molecular functions including catalytic activity. The KEGG analysis of the salt-tolerant clones showed presumed components of membrane transport, which perhaps implicated diverse metabolic pathways related to salt tolerance. Of course, whether these genes get involved in salt tolerance needs to be further experimentally demonstrated by individual cloning of each gene or random mutagenesis of these genes using a transposon assay. To maintain osmotic balance, bacteria have to alter their physiology for activating or deactivating specific enzymes or transporters to adapt to osmotic changes (Kempf and Bremer, 1998). COG functional analysis showed that the most abundant genes in salt-tolerant clones are mobilome: prophages and transposons, which can gain a nearby gene through LGT events, giving them a dominant position in a stress environment. Culligan et al. (2013) found that the salt-tolerant gene *stlA* in human gut microbiomes may belong to this family, which is only found in the human gut. Hence, gut microbes may have their own unique salt-tolerance mechanism. The COG database analysis also indicated the association of the genes with carbohydrate transport and metabolism. The results are consistent with the report of Harding et al. (2017), which stated that carbohydrate metabolism is induced by high salt conditions. The pathway enrichment analysis indicated signs that the salt-tolerant clones are involved in carbohydrate metabolism, illustrating that genes with these functions confer salt tolerance. Many genes were classified to be involved in transcription, replication, recombination, and repair. Wecker et al. (2009) studied the response of *Rhodopirellula baltica* to salt stress and found that it is associated with transcription in COG. Ivanova et al. (2011) sequenced the complete genome of the extremely halophilic *Halanaerobium praevalens* and discovered 115 genes that are in relation to replication, recombination, and repair and 150 genes connected with carbohydrate transport and metabolism.

A total of 1285 genes were detected from the salt-tolerant clones by high-throughput sequencing. Ninety-one potential genes coded proteins, such as general stress proteins, transcription regulators, trehalose-6-phosphate hydrolase, and zinc-dependent alcohol dehydrogenases, whose homologous proteins are closely related to salt tolerance. Transcription regulators are usually associated with bacterial response to various stresses (Hengge-Aronis et al., 1991; Cheville et al., 1996; Battesti et al., 2011; Hoffmann et al., 2013). Culligan et al. (2014a) subcloned and expressed the transcription regulator gene *sdtR* and found that it confers salt tolerance. Zinc-dependent alcohol dehydrogenase (Musa et al., 2008) is highly active and selective in non-water media. Trehalose-6-phosphate hydrolase may accumulate compatible solutes, which are widely used to cope with changing salinity concentrations (Roesser and Muller, 2001). Chuang et al. (2012) found that trehalose-6-phosphate hydrolase is important in the assimilation of trehalose. Moreover, the inactivating or denaturizing of proteins and cellular membranes can be prevented even in conditions such as desiccation, hypohydration, torridness, coldness, and oxidation by trehalose (Elbein et al., 2003). As we know, bacteria normally react to osmotic pressure changes in a so-called staged response, to be specific, (1) fast aggregation of K^+^; (2) succeeding synthesis or accumulating of osmoprotectant compounds (Kempf and Bremer, 1998; Sleator and Hill, 2002; Epstein, 2003; Kunte, 2006); and (3) an assisting mechanism that may include a broad scope of genes such as *htrA* gene (Sleator and Hill, 2005), GspM and EchM proteins (Kapardar et al., 2010a), ClpS protein (Kapardar et al., 2010b), *galE*, *murB*, and *mazG* genes (Culligan et al., 2012), *stlA* gene (Culligan et al., 2013), *sdtR* gene (Culligan et al., 2014a), *brpA* gene (Culligan et al., 2014b), and *TMSRP1*, *ABCTPP*, and *TLSRP1* genes (Verma et al., 2018). As such, further studies on the relations between these genes and enzymes and the microbial salt-tolerance mechanisms might prove much need.

Trehalose-6-phosphate hydrolase TRE_P2 and TRE_P3 are from *Staphylococcus warneri* according to sequence analysis. According to current findings, trehalose-6-phosphate hydrolases are identified in *E. coli* (Rimmele and Boos, 1994), *Bacillus subtilis* (Sniezko et al., 1998), *Bacillus* sp. GP16 (Karelov et al., 2003), *Bacillus licheniformis* (Chuang et al., 2012; Ong et al., 2014; Chen et al., 2015; Lin et al., 2016), *Lactobacillus acidophilus* (Duong et al., 2006), or *Klebsiella pneumoniae* (Wu et al., 2011), among which *Lactobacillus acidophilus* is a probiotic microorganism existing in the human gastrointestinal environment. At present, the largest amount of reported trehalose-6-phosphate hydrolases found were from *Bacillus*.

The biochemical characterization research of trehalose-6-phosphate hydrolase mainly focused on the substrate specificity (Rimmele and Boos, 1994), kinetic parameters (Karelov et al., 2003), structure (Ong et al., 2014), and the effect of sugar osmolytes on the refolding of trehalose-6-phosphate hydrolase (Chen et al., 2015). For example, Karelov et al. (2003) used T6P and *p*NPG as substrates to determine the *K*_*M*_ and *K*_*cat*_ values of trehalose-6-phosphate hydrolase at different pHs, temperatures, and NaCl conditions, respectively. Rimmele and Boos (1994) mainly determined the substrate specificity of trehalose-6-phosphate hydrolase. Ong et al. (2014) explored the effect of Arg201, Asn327, and Tyr365 on chloride ion binding of trehalose-6-phosphate hydrolase through mutation and biophysical analysis. Only trehalose-6-phosphate hydrolase BlTreA (Chuang et al., 2012) from *Bacillus licheniformis* have been studied in detail. However, there is no report of trehalose-6-phosphate hydrolase from the gastrointestinal tract microbial metagenome.

The cloning and expressing of *tre_P2* and *tre_P3* was successfully carried out in *E. coli*, and their products were purified and characterized. The predominant molecular mass of native rTRE_P2 and rTRE_P3 is approximately 65 kDa, indicating that the enzymes mainly exist as monomer, similar to that of most trehalose-6-phosphate hydrolases (Rimmele and Boos, 1994; Karelov et al., 2003; Chuang et al., 2012). Compared with trehalose-6-phosphate hydrolase (BlTreA) from *B. licheniformis* (Chuang et al., 2012), the optimum temperature of rTRE_P2 and rTRE_P3 is similar to that of BlTreA (30°C), and the optimum pH (7.5) is lower than that of BlTreA (pH 8.0). The activity of enzymes rose with the increasing NaCl concentration. The activity of BlTreA was elevated for roughly 3.1 times when being exposed in 100 mM NaCl, whereas the activities of rTRE_P2 and rTRE_P3 were activated by 7.4- to 35.7- and 6.4- to 35.9-fold, respectively, under the condition of 0.5–5 M NaCl. This finding may stem from an increase in solubility caused by an salt-in effect. A certain concentration of salt solution can affect the structure of proteins by salting out (decreasing protein solubility) and salting in (increasing protein solubility) (Gotsche and Dahl, 1995). Another reason may be the presence of NaCl, which causes increases in the affinity of the enzyme to the substrate (Karelov et al., 2003). Besides, the considerable amount of negatively charged acid residues dispersed on the outer layer of the protein may also play a part in it as it constituted a solvation case, preventing the protein layer from dehydrating and helping the protein adapt salinity (De Santi et al., 2016; Wang et al., 2016). Ong et al. (2014) also found out that the efficiency of BlTreA and Y365A enzymes toward *p*NPG was limited when they were desalted, maintaining less than 15.8% of the TreA activity. Given this salt-tolerance property, rTRE_P2 and rTRE_P3 are useful for reporter genes (Sniezko et al., 1998) and other salt-tolerant environments.

The experimental results show that the inhibition of rTRE_P2 and rTRE_P3 activities caused by PbCl_2_, AgNO_3_, CuSO_4_, HgCl_2_, and ZnSO_4_ probably lay in the incompatibility effect between the exogenous ions and the protein-associated cation. Chuang et al. (2012) suggested that BlTreA activity was activated by Ca^2+^ ions but was not significantly inhibited or stimulated by Co^2+^, Mn^2+^, Mg^2+^, Ni^2+^, and Fe^2+^; meanwhile, the rTRE_P2 and rTRE_P3 activities decreased to varying degrees, except for the rTRE_P2 activity that increased to 111.08% ± 6.49% and 103.90% ± 9.23% after adding Mg^2+^ and Mn^2+^, respectively. Recombinant rTRE_P2 and rTRE_P3 have dissevered trehalose-6-phosphate effectively, and glucose and glucose-6-phosphate were thus generated but do not hydrolyze trehalose. This finding is similar to that reported from *B. licheniformis* (Chuang et al., 2012) and *E. coli* (Rimmele and Boos, 1994). Hence, trehalose-6-phosphate hydrolase is probably essential for trehalose assimilation (Chuang et al., 2012) and is probably involved in the secondary response, synthesis, or accumulation of osmoprotectant compounds (Kempf and Bremer, 1998; Sleator and Hill, 2002; Epstein, 2003; Kunte, 2006).

## Conclusion

We studied the salt-tolerant genes and enzymes from the constructed gut microbial metagenomic libraries of *N. pygmaeus* and *B. frontalis* for the first time. Based on the sequencing and analysis, two salt-tolerant trehalose-6-phosphate hydrolases were identified, recombinant expressed, and characterized. TRE_P2 and TRE_P3 are the first reported salt-tolerant trehalose-6-phosphate hydrolases from the fecal microbial metagenome.

This study contributes to the limited studies of microbial salt-tolerant genes and enzymes in the gastrointestinal tract and to provide a unique genetic resource of salt-tolerant genes and enzymes for biotechnology application. Further studies of the connection between the genes and their host by transposon mutations or gene knockouts will be conducted, including identification of other enzymes involved in the trehalose metabolism. To that end, more other salt-tolerant genes and enzymes are required to fully explore the salt-tolerance mechanisms and the development and utilization of salt-tolerant genes and enzymes in gastrointestinal microorganisms.

## Data Availability Statement

The datasets generated for this study can be found in the GenBank accession numbers MN830270 and MN830271.

## Author Contributions

BX designed the study. YaY, QF, and QW screened the salt-tolerant clones. BX, YuY, JD, NH, and ZH analyzed the sequencing data and provided the technical and scientific discussion. YaY, JL, and XT performed the cloning, expression, and characterization of enzymes. The manuscript was written by YaY and revised by BX. All authors contributed to the article and approved the submitted version.

## Conflict of Interest

The authors declare that the research was conducted in the absence of any commercial or financial relationships that could be construed as a potential conflict of interest.

## Footnotes

1 ftp://ftp.ncbi.nlm.nih.gov/refseq/release/plasmid/

2 http://www.cbs.dtu.dk/services/SignalP-4.0/

3 http://espript.ibcp.fr/ESPript/ESPript/

## Supplementary Material

FIGURE S1 Functional classification of annotated transcripts by GO.

FIGURE S2 Functional classification of annotated transcripts by KEGG. **(A)** 3_1; **(B)** 1A; **(C)** 5_1_1; **(D)** 5_1_5; **(E)** 21_9A; **(F)** 24_7H; **(G)** 16_2E; **(H)** 16_2D; **(I)** 16_8H; and **(J)**: 1_2G.

FIGURE S3 SDS-PAGE analysis of the purified recombinant rTRE_P2 and rTRE_P3. M, marker proteins; 1, purified rTRE_P3; 2, unpurified rTRE_P3; 3, extracts of IPTG-induced *E. coli* BL21 (DE3) containing the empty pEASY-E2; 4, purified rTRE_P2; 5, unpurified rTRE_P2.

FIGURE S4 Hydrolysis of trehalose and trehalose-6-phosphate by rTRE_P2 and rTRE_P3. The reaction combinations were smeared on the TLC plate using a capillary tube, followed the 2 h incubation at 30°C. Buffer system mentioned above was employed to develop the combinations. **(A)** 1, glucose; 2, glucose + rTRE_P2; 3, glucose-6-phosphate; 4, glucose-6-phosphate + rTRE_P2; 5, trehalose; 6, trehalose + rTRE_P2; 7, trehalose-6-phosphate; 8, trehalose-6-phosphate + rTRE_P2. **(B)** 1, glucose; 2, glucose + rTRE_P3; 3, glucose-6-phosphate; 4, glucose-6-phosphate + rTRE_P3; 5, trehalose; 6, trehalose + rTRE_P3; 7, trehalose-6-phosphate; and 8, trehalose-6-phosphate + rTRE_P3.

TABLE S1 Statistics of the trimmed sequences.

TABLE S2 Detection of putative salt tolerance enzymes and genes of the salt tolerance clones.
