# Supplementary figures and images for: Molecular and Biochemical Characterization of Salt-Tolerant Trehalose-6-Phosphate Hydrolases Identified by Screening and Sequencing Salt-Tolerant Clones From the Metagenomic Library of the Gastrointestinal Tract
Source: Front Microbiol. 2020 Jul 7;11:1466. doi: 10.3389/fmicb.2020.01466 (PMC7358406; doi:10.3389/fmicb.2020.01466)

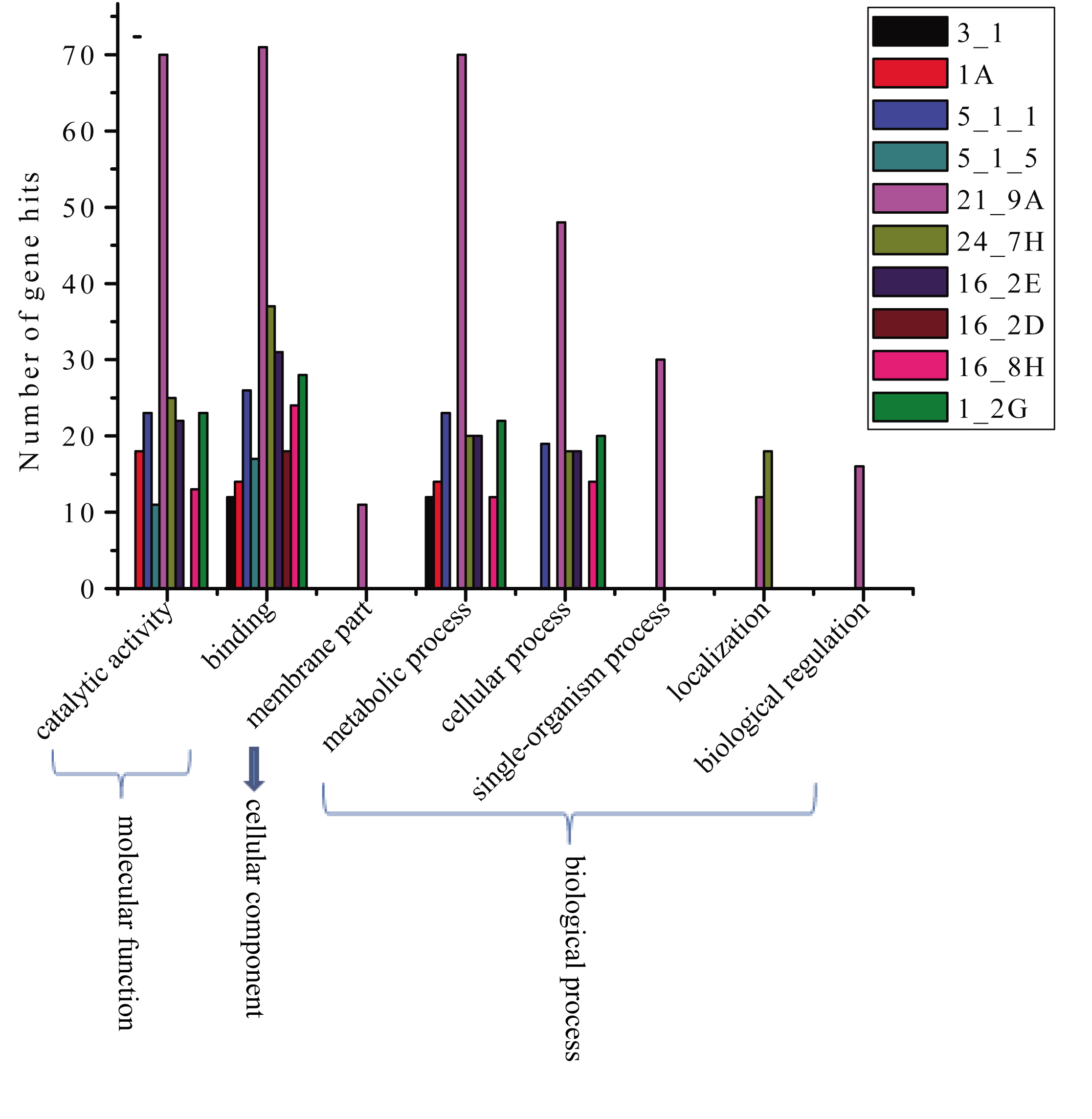

Supplement: FIGURE S1 — Functional classification of annotated transcripts by GO. [file Image_1.TIF]

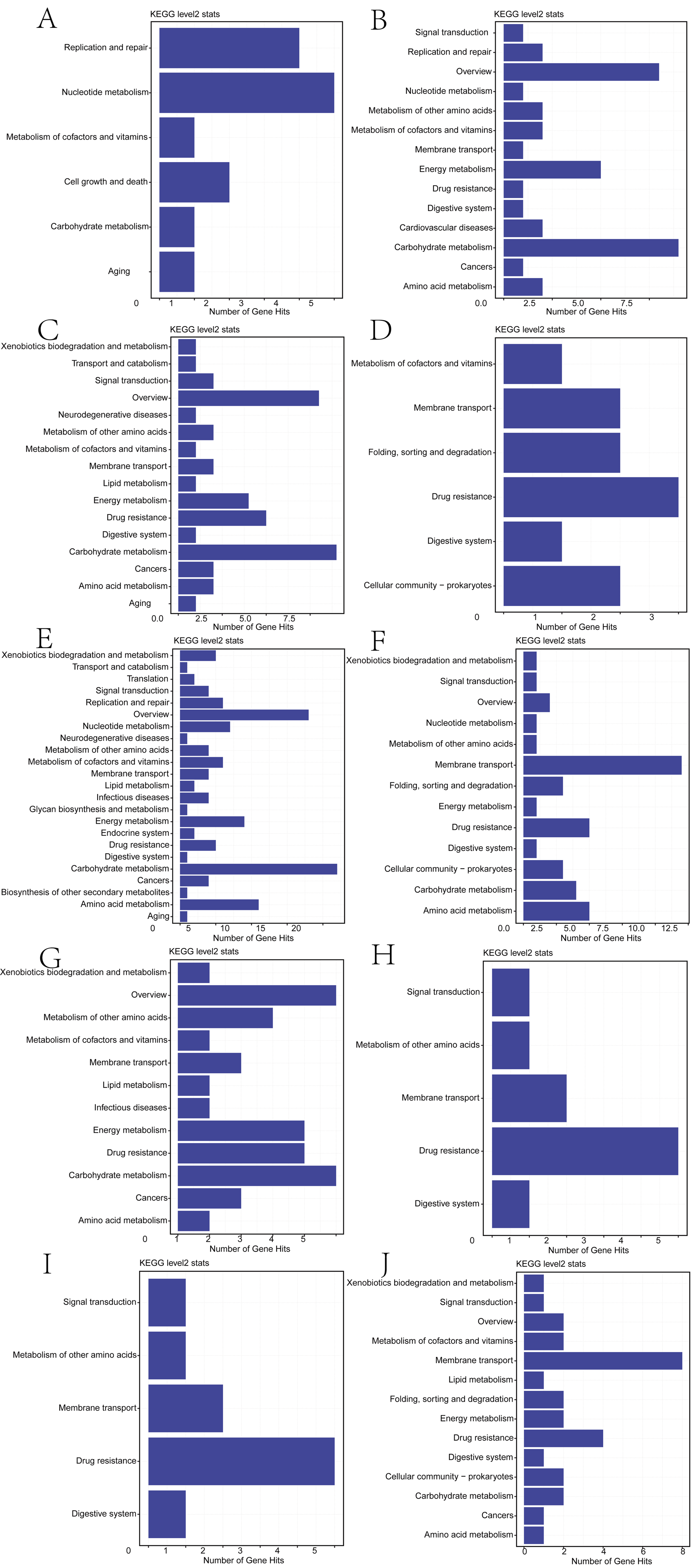

Supplement: FIGURE S2 — Functional classification of annotated transcripts by KEGG. (A) 3_1; (B) 1A; (C) 5_1_1; (D) 5_1_5; (E) 21_9A; (F) 24_7H; (G) 16_2E; (H) 16_2D; (I) 16_8H; and (J): 1_2G. [file Image_2.TIF]

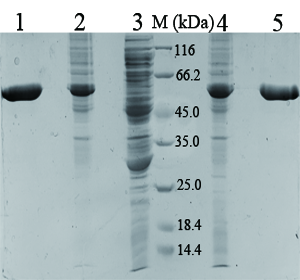

Supplement: FIGURE S3 — SDS-PAGE analysis of the purified recombinant rTRE_P2 and rTRE_P3. M, marker proteins; 1, purified rTRE_P3; 2, unpurified rTRE_P3; 3, extracts of IPTG-induced E. coli BL21 (DE3) containing the empty pEASY-E2; 4, purified rTRE_P2; 5, unpurified rTRE_P2. [file Image_3.TIF]

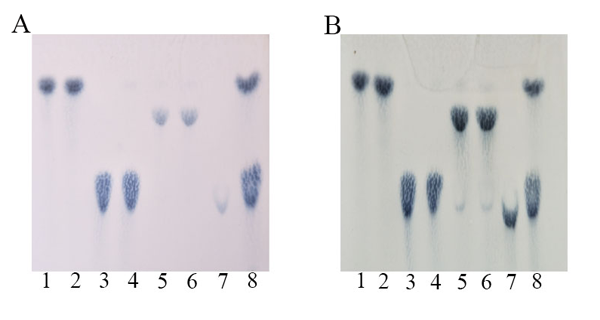

Supplement: FIGURE S4 — Hydrolysis of trehalose and trehalose-6-phosphate by rTRE_P2 and rTRE_P3. The reaction combinations were smeared on the TLC plate using a capillary tube, followed the 2 h incubation at 30°C. Buffer system mentioned above was employed to develop the combinations. (A) 1, glucose; 2, glucose + rTRE_P2; 3, glucose-6-phosphate; 4, glucose-6-phosphate + rTRE_P2; 5, trehalose; 6, trehalose + rTRE_P2; 7, trehalose-6-phosphate; 8, trehalose-6-phosphate + rTRE_P2. (B) 1, glucose; 2, glucose + rTRE_P3; 3, glucose-6-phosphate; 4, glucose-6-phosphate + rTRE_P3; 5, trehalose; 6, trehalose + rTRE_P3; 7, trehalose-6-phosphate; and 8, trehalose-6-phosphate + rTRE_P3. [file Image_4.TIF]
