# Supplementary material for: Molecular and Biochemical Characterization of Salt-Tolerant Trehalose-6-Phosphate Hydrolases Identified by Screening and Sequencing Salt-Tolerant Clones From the Metagenomic Library of the Gastrointestinal Tract
Source: Front Microbiol. 2020 Jul 7;11:1466. doi: 10.3389/fmicb.2020.01466 (PMC7358406; doi:10.3389/fmicb.2020.01466)
Supplement: TABLE S1 — Statistics of the trimmed sequences. [file Table_1.DOCX]

| **Table S1 Statistics of the trimmed sequences** | | | | | | | | | | | |
| --- | --- | --- | --- | --- | --- | --- | --- | --- | --- | --- | --- |
| Sample ID | Raw Reads | Raw Bases  (bp) | Clean Reads | Clean  Reads  (bp) | The total number of scaffolds | The total length of scaffold (bp) | The mean length of scaffold  (bp) | N50 length  (bp) | GC% | The length  of gaps | The number of gaps |
| 3_1 | 12532608 | 1879891200 | 10191538 | 1528200686 | 4 | 82469 | 20617.25 | 76983 | 4 | 82469 | 20617.25 |
| 1A | 9211958 | 1381793700 | 7752664 | 1162281606 | 11 | 61348 | 5577.09 | 2452 | 11 | 61348 | 5577.09 |
| 5_1_1 | 8629676 | 1294451400 | 8364350 | 1254392993 | 29 | 105297 | 3630.93 | 1463 | 29 | 105297 | 3630.93 |
| 5_1_5 | 10242916 | 1536437400 | 9871672 | 1477284362 | 14 | 74067 | 5290.50 | 3712 | 14 | 74067 | 5290.50 |
| 21_9A | 9901198 | 1485179700 | 9631280 | 1442996282 | 55 | 310265 | 5641.18 | 2777 | 55 | 310265 | 5641.18 |
| 16_7A | 10061678 | 1509251700 | 9851812 | 1477356792 | 18 | 157154 | 8730.78 | 3291 | 18 | 157154 | 8730.78 |
| 16_2E | 13453366 | 2018004900 | 13182414 | 1976778181 | 36 | 130315 | 3619.86 | 1519 | 36 | 130315 | 3619.86 |
| 16_2D | 7649476 | 1147421400 | 7403920 | 1110345849 | 15 | 69124 | 4608.27 | 2528 | 15 | 69124 | 4608.27 |
| 16_8H | 9697488 | 1454623200 | 9395602 | 1408555496 | 16 | 99436 | 6214.75 | 4666 | 16 | 99436 | 6214.75 |
| 1_2G | 9529594 | 1429439100 | 8888506 | 1323587384 | 47 | 132352 | 2816.00 | 1389 | 47 | 132352 | 2816.00 |
