# Supplementary material for: Molecular and Biochemical Characterization of Salt-Tolerant Trehalose-6-Phosphate Hydrolases Identified by Screening and Sequencing Salt-Tolerant Clones From the Metagenomic Library of the Gastrointestinal Tract
Source: Front Microbiol. 2020 Jul 7;11:1466. doi: 10.3389/fmicb.2020.01466 (PMC7358406; doi:10.3389/fmicb.2020.01466)
Supplement: TABLE S2 — Detection of putative salt tolerance enzymes and genes of the salt tolerance clones. [file Table_2.docx]

| **Table S2 Detection of putative salt tolerance enzymes and genes of the salt tolerance clones** | | | | | | |  |
| --- | --- | --- | --- | --- | --- | --- | --- |
| Query_id | source | Amino  acid  length | Identity | Function annotation in Nr | Stress tolerance  of homologous  genes | Reference | |
| Plasmid2_Scaffold04_orf19 | 1A | 308 | 96.1 | UDP-glucose 4-epimerase | ++ | (Nguyen et al., 2004; Culligan et al., 2012) | |
| Plasmid2_Scaffold05_orf25 | 1A | 172 | 100 | general stress protein | ++ | (Maul et al., 1995; Kapardar et al., 2010) | |
| Plasmid10_plasmid02_8 | 1_2G | 172 | 100 |  |  |  |  |
| Plasmid10_plasmid13_3 | 1_2G | 137 | 100 | universal stress protein | + | (Kvint et al., 2003) | |
| Plasmid1_Scaffold01_orf46 | 3-1 | 72 | 100 | transcriptional regulator | ++ | (Hengge-Aronis et al., 1991; Cheville et al., 1996; Battesti et al., 2011; Hoffmann et al., 2013) | |
| Plasmid1_Scaffold01_orf105 | 3-1 | 82 | 98.8 |  |  |  |  |
| Plasmid2_Scaffold05_orf28 | 1A | 114 | 99.1 |  |  |  |  |
| Plasmid2_Scaffold05_orf34 | 1A | 110 | 99.1 |  |  |  |  |
| Plasmid2_Scaffold07_orf55 | 1A | 91 | 98.9 |  |  |  |  |
| Plasmid3_plasmid01_5 | 5_1_1 | 110 | 99.1 |  |  |  |  |
| Plasmid3_plasmid03_5 | 5_1_1 | 93 | 100 |  |  |  |  |
| Plasmid3_plasmid15_1 | 5_1_1 | 127 | 91.2 |  |  |  |  |
| Plasmid4_plasmid01_26 | 21_9A | 112 | 100 |  |  |  |  |
| Plasmid4_plasmid06_11 | 21_9A | 347 | 99.4 |  |  |  |  |
| Plasmid4_plasmid13_5 | 21_9A | 91 | 98.9 |  |  |  |  |
| Plasmid6_plasmid01_70 | 16_7A | 46 | 45.7 |  |  |  |  |
| Plasmid6_plasmid02_23 | 16_7A | 91 | 98.9 |  |  |  |  |
| Plasmid7_plasmid03_8 | 16_2E | 112 | 100 |  |  |  |  |
| Plasmid8_plasmid01_5 | 16_2D | 91 | 98.9 |  |  |  |  |
| Plasmid9_plasmid02_23 | 16_8H | 91 | 98.9 |  |  |  |  |
| Plasmid10_plasmid02_10 | 1_2G | 117 | 100 |  |  |  |  |
| Plasmid10_plasmid05_5 | 1_2G | 91 | 98.9 |  |  |  |  |
| Plasmid10_plasmid12_3 | 1_2G | 188 | 100 |  |  |  |  |
| Plasmid11_plasmid01_10 | 5_1_5 | 91 | 98.9 |  |  |  |  |
| Plasmid11_plasmid01_21 | 5_1_5 | 188 | 100 |  |  |  |  |
| Plasmid2_Scaffold04_orf5 | 1A | 244 | 99.6 | GntR family transcriptional regulator |  |  |  |
| Plasmid4_plasmid14_2 | 21_9A | 241 | 100 |  |  |  |  |
| Plasmid2_Scaffold04_orf11 | 1A | 220 | 100 | LysR family transcriptional regulator |  |  |  |
| Plasmid3_plasmid02_2 | 5_1_1 | 115 | 97.4 | ArsR family transcriptional regulator |  |  |  |
| Plasmid4_plasmid01_33 | 21_9A | 48 | 100 |  |  |  |  |
| Plasmid4_plasmid02_16 | 21_9A | 106 | 100 |  |  |  |  |
| Plasmid4_plasmid02_21 | 21_9A | 104 | 100 |  |  |  |  |
| Plasmid7_plasmid03_1 | 16_2E | 48 | 100 |  |  |  |  |
| Plasmid10_plasmid09_6 | 1_2G | 48 | 100 |  |  |  |  |
| Plasmid3_plasmid02_15 | 5_1_1 | 100 | 98 | PadR family transcriptional regulator |  |  |  |
| Plasmid10_plasmid02_4 | 1_2G | 100 | 100 |  |  |  |  |
| Plasmid4_plasmid02_4 | 21_9A | 85 | 100 | CopG family transcriptional regulator |  |  |  |
| Plasmid9_plasmid04_4 | 16_8H | 85 | 100 |  |  |  |  |
| Plasmid4_plasmid02_7 | 21_9A | 82 | 67.1 | AbrB family transcriptional regulator |  |  |  |
| Plasmid4_plasmid03_34 | 21_9A | 82 | 96.3 |  |  |  |  |
| Plasmid6_plasmid03_4 | 16_7A | 89 | 95.5 |  |  |  |  |
| Plasmid7_plasmid01_3 | 16_2E | 89 | 95.5 |  |  |  |  |
| Plasmid8_plasmid02_3 | 16_2D | 82 | 96.3 |  |  |  |  |
| Plasmid9_plasmid01_34 | 16_8H | 82 | 96.3 |  |  |  |  |
| Plasmid4_plasmid04_10 | 21_9A | 329 | 97.6 | LacI family transcriptional regulator |  |  |  |
| Plasmid6_plasmid01_63 | 16_7A | 329 | 97.6 |  |  |  |  |
| Plasmid4_plasmid04_18 | 21_9A | 190 | 100 | TetR family transcriptional regulator |  |  |  |
| Plasmid4_plasmid04_21 | 21_9A | 191 | 99.5 |  |  |  |  |
| Plasmid4_plasmid05_4 | 21_9A | 194 | 100 |  |  |  |  |
| Plasmid6_plasmid01_52 | 16_7A | 191 | 99.5 |  |  |  |  |
| Plasmid6_plasmid01_55 | 16_7A | 190 | 100 |  |  |  |  |
| Plasmid4_plasmid06_2 | 21_9A | 268 | 99.6 | RpiR family transcriptional regulator |  |  |  |
| Plasmid4_plasmid11_2 | 21_9A | 172 | 96.5 | MarR family transcriptional regulator |  |  |  |
| Plasmid7_plasmid07_6 | 16_2E | 172 | 96.5 |  |  |  |  |
| Plasmid9_plasmid03_20 | 16_8H | 172 | 96.5 |  |  |  |  |
| Plasmid6_plasmid01_14 | 16_7A | 158 | 100 | rgR family transcriptional regulator |  |  |  |
| Plasmid6_plasmid01_19 | 16_7A | 237 | 100 | Crp/Fnr family transcriptional regulator |  |  |  |
| Plasmid7_plasmid11_3 | 16_2E | 91 | 100 | XRE family transcriptional regulator |  |  |  |
| Plasmid2_Scaffold04_orf21 | 1A | 66 | 100 | cold-shock protein | + | (Ermolenko and Makhatadze, 2002) | |
| Plasmid2_Scaffold05_orf23 | 1A | 310 | 100 | zinc-dependent alcohol dehydrogenase | ++ | (Musa et al., 2008) | |
| Plasmid3_plasmid02_13 | 5_1_1 | 383 | 99.2 | alcohol dehydrogenase |  |  |  |
| Plasmid4_plasmid01_25 | 21_9A | 61 | 100 |  |  |  |  |
| Plasmid7_plasmid03_9 | 16_2E | 64 | 100 |  |  |  |  |
| Plasmid10_plasmid02_6 | 1_2G | 383 | 99.2 |  |  |  |  |
| Plasmid2_Scaffold05_orf38 | 1A | 468 | 100 | 6-phosphogluconate dehydrogenase | + | (Cairns et al., 2011) | |
| Plasmid3_plasmid01_9 | 5_1_1 | 468 | 100 |  |  |  |  |
| Plasmid4_plasmid01_29 | 21_9A | 468 | 100 |  |  |  |  |
| Plasmid7_plasmid03_5 | 16_2E | 468 | 100 |  |  |  |  |
| Plasmid2_Scaffold05_orf39 | 1A | 486 | 99.8 | glucose-6-phosphate 1-dehydrogenase | ++ | (Van Assche et al., 1988; Nemoto and Sasakuma, 2000; Huang et al., 2003) | |
| Plasmid3_plasmid01_10 | 5_1_1 | 486 | 100 |  |  |  |  |
| Plasmid4_plasmid01_28 | 21_9A | 490 | 97.6 |  |  |  |  |
| Plasmid7_plasmid03_6 | 16_2E | 490 | 99.6 |  |  |  |  |
| Plasmid2_Scaffold04_orf6 | 1A | 545 | 99.1 | trehalose-6-phosphate hydrolase | ++ | (Elbein et al., 2003; Chuang et al., 2012) | |
| Plasmid3_plasmid05_4 | 5_1_1 | 545 | 99.3 |  |  |  |  |
| Plasmid4_plasmid14_1 | 21_9A | 245 | 100 |  |  |  |  |
| Plasmid2_Scaffold07_orf57 | 1A | 151 | 98.7 | cation transporter | ++ | (Yaish et al., 2017) | |
| Plasmid3_plasmid03_7 | 5_1_1 | 310 | 100 |  |  |  |  |
| Plasmid4_plasmid13_3 | 21_9A | 313 | 100 |  |  |  |  |
| Plasmid6_plasmid02_25 | 16_7A | 310 | 100 |  |  |  |  |
| Plasmid7_plasmid18_3 | 16_2E | 103 | 100 |  |  |  |  |
| Plasmid8_plasmid01_3 | 16_2D | 313 | 100 |  |  |  |  |
| Plasmid9_plasmid02_25 | 16_8H | 313 | 100 |  |  |  |  |
| Plasmid10_plasmid05_7 | 1_2G | 313 | 100 |  |  |  |  |
| Plasmid11_plasmid01_12 | 5_1_5 | 313 | 100 |  |  |  |  |
| Plasmid3_plasmid01_3 | 5_1_1 | 320 | 99.4 | ATPase AAA | ++ | (Guo et al., 2014) | |
| Plasmid10_plasmid01_12 | 1_2G | 885 | 61.2 |  |  |  |  |
| Plasmid11_plasmid02_3 | 5_1_5 | 885 | 61.2 |  |  |  |  |
| Plasmid3_plasmid03_4 | 5_1_1 | 795 | 99.9 | ATPase P |  |  |  |
| Plasmid4_plasmid13_7 | 21_9A | 331 | 100 |  |  |  |  |
| Plasmid6_plasmid02_22 | 16_7A | 795 | 100 |  |  |  |  |
| Plasmid8_plasmid01_6 | 16_2D | 795 | 100 |  |  |  |  |
| Plasmid9_plasmid02_22 | 16_8H | 795 | 100 |  |  |  |  |
| Plasmid10_plasmid05_4 | 1_2G | 616 | 100 |  |  |  |  |
| Plasmid3_plasmid02_1 | 5_1_1 | 663 | 87.2 | catalase | ++ | (Gebicka and Didik, 2009) | |
| Plasmid3_plasmid02_3 | 5_1_1 | 205 | 98 | cadmium transporter | + | (Liu et al., 2009; Thevenod and Lee, 2013; Rani et al., 2014; Yang and Shu, 2015) | |
| Plasmid3_plasmid02_12 | 5_1_1 | 753 | 99.9 | daunorubicin resistance protein DrrC | + | (Furuya and Hutchinson, 1998; Prija and Prasad, 2017) | |
| Plasmid4_plasmid01_27 | 21_9A | 752 | 99.3 |  |  |  |  |
| Plasmid7_plasmid03_7 | 16_2E | 752 | 99.3 |  |  |  |  |
| Plasmid10_plasmid02_7 | 1_2G | 753 | 99.9 |  |  |  |  |
| Plasmid3_plasmid02_16 | 5_1_1 | 133 | 97 | arsenate reductase | ++ | (Kumari and Jagadevan, 2016) | |
| Plasmid4_plasmid02_23 | 21_9A | 131 | 100 |  |  |  |  |
| Plasmid3_plasmid02_22 | 5_1_1 | 241 | 100 | beta-lactamase | + | (Livermore, 1995) | |
| Plasmid3_plasmid05_3 | 5_1_1 | 286 | 99.7 |  |  |  |  |
| Plasmid9_plasmid02_1 | 16_8H | 281 | 100 | BlaZ family class A beta-lactamase |  |  |  |
| Plasmid10_plasmid11_1 | 1_2G | 281 | 100 |  |  |  |  |
| Plasmid11_plasmid01_15 | 5_1_5 | 281 | 100 |  |  |  |  |
| Plasmid4_plasmid01_13 | 21_9A | 381 | 85.3 | nitric oxide dioxygenase | + | (Oleksiewicz et al., 2011) | |
| Plasmid4_plasmid02_11 | 21_9A | 241 | 100 |  |  |  |  |
| Plasmid4_plasmid02_12 | 21_9A | 94 | 100 |  |  |  |  |
| Plasmid4_plasmid07_6 | 21_9A | 294 | 100 | heat shock protein Hsp33 | + | (Wholey and Jakob, 2012) | |
| Plasmid4_plasmid13_4 | 21_9A | 101 | 100 | glutaredoxin | + | (Shen et al., 2018) | |
| Plasmid6_plasmid02_24 | 16_7A | 101 | 100 |  |  |  |  |
| Plasmid7_plasmid18_2 | 16_2E | 101 | 100 |  |  |  |  |
| Plasmid8_plasmid01_4 | 16_2D | 101 | 100 |  |  |  |  |
| Plasmid9_plasmid02_24 | 16_8H | 101 | 100 |  |  |  |  |
| Plasmid10_plasmid05_6 | 1_2G | 101 | 99 |  |  |  |  |
| Plasmid11_plasmid01_11 | 5_1_5 | 101 | 100 |  |  |  |  |
| Plasmid6_plasmid01_15 | 16_7A | 411 | 100 | arginine deiminase | + | (Somani and Chaskar, 2018) | |
| “++” means that homologous genes of unigene exhibited tolerance against salt stress；“+” represents that homologous genes of  unigene tolerated other stress. | | | | | | | |

REFERENCES

Battesti, A., Majdalani, N., and Gottesman, S. (2011). The RpoS-mediated general stress response in *Escherichia coli*. *Annu Rev Microbiol* 65**,** 189-213. doi: 10.1146/annurev-micro-090110-102946.

Cairns, R.A., Harris, I.S., and Mak, T.W. (2011). Regulation of cancer cell metabolism. *Nat Rev Cancer* 11(2)**,** 85-95. doi: 10.1038/nrc2981.

Cheville, A.M., Arnold, K.W., Buchrieser, C., Cheng, C.M., and Kaspar, C.W. (1996). rpoS regulation of acid, heat, and salt tolerance in *Escherichia coli* O157:H7. *Appl Environ Microbiol* 62(5)**,** 1822-1824.

Chuang, T.T., Ong, P.L., Wang, T.F., Huang, H.B., Chi, M.C., and Lin, L.L. (2012). Molecular characterization of a novel trehalose-6-phosphate hydrolase, TreA, from *Bacillus licheniformis*. *Int J Biol Macromol* 50(3)**,** 459-470. doi: 10.1016/j.ijbiomac.2012.01.011.

Culligan, E.P., Sleator, R.D., Marchesi, J.R., and Hill, C. (2012). Functional metagenomics reveals novel salt tolerance loci from the human gut microbiome. *Isme j* 6(10)**,** 1916-1925. doi: 10.1038/ismej.2012.38.

Elbein, A.D., Pan, Y.T., Pastuszak, I., and Carroll, D. (2003). New insights on trehalose: a multifunctional molecule. *Glycobiology* 13(4)**,** 17r-27r. doi: 10.1093/glycob/cwg047.

Ermolenko, D.N., and Makhatadze, G.I. (2002). Bacterial cold-shock proteins. *Cell Mol Life Sci* 59(11)**,** 1902-1913.

Furuya, K., and Hutchinson, C.R. (1998). The DrrC protein of *Streptomyces peucetius*, a UvrA-like protein, is a DNA-binding protein whose gene is induced by daunorubicin. *FEMS Microbiol Lett* 168(2)**,** 243-249. doi: 10.1111/j.1574-6968.1998.tb13280.x.

Gebicka, L., and Didik, J. (2009). Catalytic scavenging of peroxynitrite by catalase. *J Inorg Biochem* 103(10)**,** 1375-1379. doi: 10.1016/j.jinorgbio.2009.07.011.

Guo, M., Wang, R., Wang, J., Hua, K., Wang, Y., Liu, X., et al. (2014). ALT1, a Snf2 family chromatin remodeling ATPase, negatively regulates alkaline tolerance through enhanced defense against oxidative stress in rice. *PLoS One* 9(12)**,** e112515. doi: 10.1371/journal.pone.0112515.

Hengge-Aronis, R., Klein, W., Lange, R., Rimmele, M., and Boos, W. (1991). Trehalose synthesis genes are controlled by the putative sigma factor encoded by rpoS and are involved in stationary-phase thermotolerance in *Escherichia coli*. *J Bacteriol* 173(24)**,** 7918-7924.

Hoffmann, R.F., McLernon, S., Feeney, A., Hill, C., and Sleator, R.D. (2013). A single point mutation in the listerial betL sigma(A)-dependent promoter leads to improved osmo- and chill-tolerance and a morphological shift at elevated osmolarity. *Bioengineered* 4(6)**,** 401-407. doi: 10.4161/bioe.24094.

Huang, J., Zhang, H., Wang, J., and Yang, J. (2003). Molecular cloning and characterization of rice 6-phosphogluconate dehydrogenase gene that is up-regulated by salt stress. *Mol Biol Rep* 30(4)**,** 223-227.

Kapardar, R.K., Ranjan, R., Grover, A., Puri, M., and Sharma, R. (2010). Identification and characterization of genes conferring salt tolerance to *Escherichia coli* from pond water metagenome. *Bioresour Technol* 101(11)**,** 3917-3924. doi: 10.1016/j.biortech.2010.01.017.

Kumari, N., and Jagadevan, S. (2016). Genetic identification of arsenate reductase and arsenite oxidase in redox transformations carried out by arsenic metabolising prokaryotes - A comprehensive review. *Chemosphere* 163**,** 400-412. doi: 10.1016/j.chemosphere.2016.08.044.

Kvint, K., Nachin, L., Diez, A., and Nystrom, T. (2003). The bacterial universal stress protein: function and regulation. *Curr Opin Microbiol* 6(2)**,** 140-145.

Liu, J., Qu, W., and Kadiiska, M.B. (2009). Role of oxidative stress in cadmium toxicity and carcinogenesis. *Toxicol Appl Pharmacol* 238(3)**,** 209-214. doi: 10.1016/j.taap.2009.01.029.

Livermore, D.M. (1995). beta-Lactamases in laboratory and clinical resistance. *Clin Microbiol Rev* 8(4)**,** 557-584.

Maul, B., Volker, U., Riethdorf, S., Engelmann, S., and Hecker, M. (1995). sigma B-dependent regulation of gsiB in response to multiple stimuli in *Bacillus subtilis*. *Mol Gen Genet* 248(1)**,** 114-120.

Musa, M.M., Ziegelmann-Fjeld, K.I., Vieille, C., and Phillips, R.S. (2008). Activity and selectivity of W110A secondary alcohol dehydrogenase from *Thermoanaerobacter* *ethanolicus* in organic solvents and ionic liquids: mono- and biphasic media. *Org Biomol Chem* 6(5)**,** 887-892. doi: 10.1039/b717120j.

Nemoto, Y., and Sasakuma, T. (2000). Specific expression of glucose-6-phosphate dehydrogenase (G6PDH) gene by salt stress in wheat (Triticum aestivum L.). *Plant Sci* 158(1-2)**,** 53-60.

Nguyen, T.T., Klueva, N., Chamareck, V., Aarti, A., Magpantay, G., Millena, A.C., et al. (2004). Saturation mapping of QTL regions and identification of putative candidate genes for drought tolerance in rice. *Mol Genet Genomics* 272(1)**,** 35-46. doi: 10.1007/s00438-004-1025-5.

Oleksiewicz, U., Liloglou, T., Field, J.K., and Xinarianos, G. (2011). Cytoglobin: biochemical, functional and clinical perspective of the newest member of the globin family. *Cell Mol Life Sci* 68(23)**,** 3869-3883. doi: 10.1007/s00018-011-0764-9.

Prija, F., and Prasad, R. (2017). DrrC protein of Streptomyces peucetius removes daunorubicin from intercalated dnrI promoter. *Microbiol Res* 202**,** 30-35. doi: 10.1016/j.micres.2017.05.002.

Rani, A., Kumar, A., Lal, A., and Pant, M. (2014). Cellular mechanisms of cadmium-induced toxicity: a review. *Int J Environ Health Res* 24(4)**,** 378-399. doi: 10.1080/09603123.2013.835032.

Shen, Z.J., Zhang, S.D., Liu, Y.J., Liu, X.M., Li, Z., Zhang, Q.W., et al. (2018). Functional analysis by RNAi of an glutaredoxin gene in *Helicoverpa armigera*. *J Insect Physiol* 106(Pt 2)**,** 98-105. doi: 10.1016/j.jinsphys.2017.10.011.

Somani, R.R., and Chaskar, P.K. (2018). Arginine Deiminase Enzyme Evolving as a Potential Antitumor Agent. *Mini Rev Med Chem* 18(4)**,** 363-368. doi: 10.2174/1389557516666160817102701.

Thevenod, F., and Lee, W.K. (2013). Cadmium and cellular signaling cascades: interactions between cell death and survival pathways. *Arch Toxicol* 87(10)**,** 1743-1786. doi: 10.1007/s00204-013-1110-9.

Van Assche, F., Cardinaels, C., and Clijsters, H. (1988). Induction of enzyme capacity in plants as a result of heavy metal toxicity: dose-response relations in *Phaseolus vulgaris* L., treated with zinc and cadmium. *Environ Pollut* 52(2)**,** 103-115.

Wholey, W.Y., and Jakob, U. (2012). Hsp33 confers bleach resistance by protecting elongation factor Tu against oxidative degradation in Vibrio cholerae. *Mol Microbiol* 83(5)**,** 981-991. doi: 10.1111/j.1365-2958.2012.07982.x.

Yaish, M.W., Patankar, H.V., Assaha, D.V.M., Zheng, Y., Al-Yahyai, R., and Sunkar, R. (2017). Genome-wide expression profiling in leaves and roots of date palm (Phoenix dactylifera L.) exposed to salinity. *BMC Genomics* 18(1)**,** 246. doi: 10.1186/s12864-017-3633-6.

Yang, H., and Shu, Y. (2015). Cadmium transporters in the kidney and cadmium-induced nephrotoxicity. *Int J Mol Sci* 16(1)**,** 1484-1494. doi: 10.3390/ijms16011484.
